# Quantitative proteomics identified a novel invasion biomarker associated with EMT in pituitary adenomas

**DOI:** 10.3389/fendo.2023.1137648

**Published:** 2023-03-03

**Authors:** Yu Zhang, Lei Li, Xin Ma, Chenan Liu, Gemingtian Liu, Zhixu Bie, Zhijun Yang, Pinan Liu

**Affiliations:** ^1^ Department of Neurosurgery, Beijing Tiantan Hospital, Capital Medical University, Beijing, China; ^2^ Central Laboratory, Capital Medical University, Beijing, China; ^3^ Department of Gastrointestinal Surgery, Beijing Shijitan Hospital, Capital Medical University, Beijing, China; ^4^ Department of Neural Reconstruction, Beijing Key Laboratory of Central Nervous System Injury, Beijing Neurosurgical Institute, Capital Medical University, Beijing, China

**Keywords:** pituitary adenomas, invasion, EMT, SLC2A1, biomarker

## Abstract

**Background:**

Complete resection of invasive pituitary adenoma is usually difficult, resulting in a high recurrence rate. Therefore, it is needed to find potential diagnostic markers and therapeutic targets for invasive pituitary adenoma.

**Methods:**

We collected samples from patients with invasive and non-invasive pituitary adenomas from Beijing Tiantan Hospital for protein extraction and quantitative analysis. We identified differential proteins (DEPs) by differential analysis of the two groups. The intersection of differential proteins related to invasion and epithelial-mesenchymal transition (EMT) in the GeneCards database was identified as EMT-DEPs. The protein network of EMT-DEPs was analyzed using the STRING database and Cytoscape software, and the hub EMT-DEPs were obtained by the MCC algorithm of the cytoHubba plugin. Correlation analysis was used to obtain the interpairing proteins among EMT-DEPs, and core EMT-DEPs were identified based on the number of paired proteins. The Venn program was used to identify the intersection of hub EMT-DEPs and core EMT-DEPs as key EMT-DEPs. Finally, a series of analyses plus experiments were used to verify the correlation of the target protein with invasion and EMT in pituitary adenoma.

**Results:**

Quantitative comparison of proteins between invasive and non-invasive pituitary adenomas indicated 833 differential proteins. The overlaps of EMT-related proteins and differential proteins consisted of 46 EMT-DEPs. There were 6 intersections between the hub EMT-DEPs and core EMT-DEPs. Using quantitative protein data and GSE169498 chip, we found that solute carrier family 2 member 1 (SLC2A1) was our target protein. SLC2A1 was significantly correlated with the invasiveness of pituitary adenoma, and the ROC curve was satisfactory. The functions and pathways of SLC2A1 and paired protein enrichment were closely linked to the EMT. Consistently, SLC2A1 expression was significantly and positively correlated with the expression of classical markers of EMT. The final experiment revealed that SLC2A1 was significantly upregulated in invasive pituitary adenoma.

**Conclusion:**

SLC2A1 is significantly upregulated in invasive pituitary adenoma with satisfactory predictive value. It may regulate EMT. It may be a potential diagnostic marker for invasive pituitary adenoma.

## Introduction

Pituitary adenomas originate from endocrine cells in the adenohypophysis. They are mostly benign; however, some types of pituitary adenoma show unpredictable invasiveness known as invasive pituitary adenomas (1–3). Invasive pituitary adenomas are rare, but they can rapidly progress and invade surrounding tissues. They have a high risk of recurrence and may resist the standard therapy. Therefore, they have been classified as “high-risk” pituitary adenoma by the World Health Organization in 2017 (4, 5). Surgical resection of invasive pituitary adenoma is difficult. Whether it is performed through transsphenoidal or transcranial surgery, it requires the surgeon to make reasonable adjustments or even expand the standard protocol according to the tumor condition. Still, complete removal of the tumor cannot be achieved, which poses a higher risk of recurrence. Therefore, surgical treatment can mainly relieve the symptoms of patients and may temporarily control the disease. Patients usually need to receive radiotherapy, medications, and even chemotherapy after surgery. However, the tumor continues to grow even after all of these modalities (6). In recent years, studies on invasive pituitary adenomas have brought some progresses. It has been suggested that histological features such as Ki 67 ≥ 3%, increased mitosis, and increased p53 expression can be used to predict the invasiveness of pituitary adenomas and to find new therapeutic targets, but their predictive value and accuracy are still controversial (1, 7). Epithelial-mesenchymal transition (EMT) can promote invasion and metastasis by inducing mesenchymal properties, including anti-anoikis properties and the ability to migrate and invade the surrounding tissues (8). We, therefore, sought to explore the molecular markers for the invasiveness of pituitary adenoma, which may regulate EMT.

## Materials and methods

**Figure 1:**
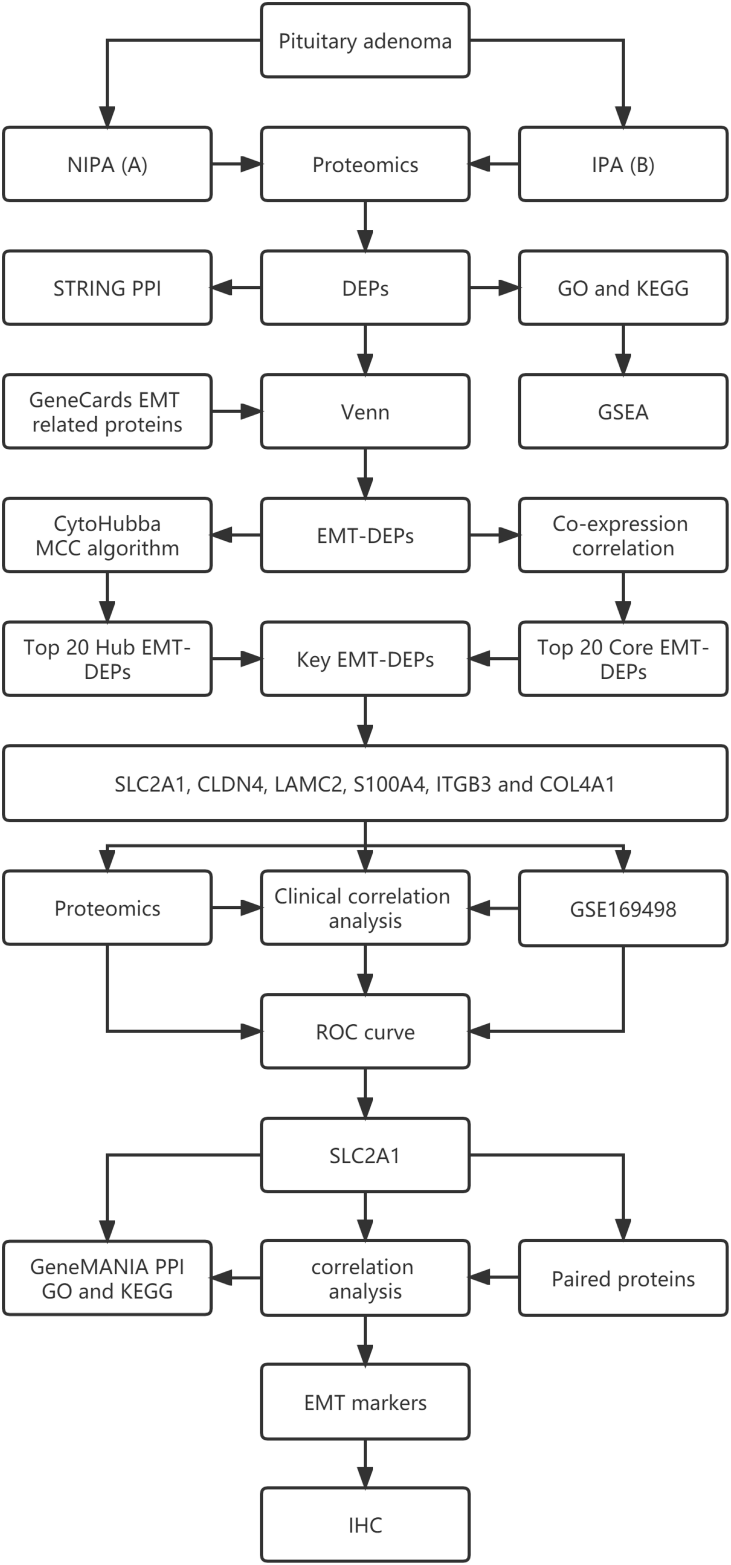
The flow chart of this study.

**Figure 1** shows the workflow of this study.

### Tissue samples

Tissue samples were obtained from 19 patients who attended the Neurosurgery Department of Beijing Tiantan Hospital for resection of pituitary adenoma. The protocol of this study was approved by the Ethics Committee of Beijing Tiantan Hospital, and all patients signed the informed consent form. Postoperative tissue samples were examined at the Department of Pathology, Beijing Tiantan Hospital, and all histological assessments confirmed pituitary adenoma. Invasive and non-invasive adenomas were identified using imaging and pathology reports. Ten invasive samples were silent corticotroph adenomas, and 9 non-invasive samples were follicle-stimulating hormone (FSH)-secreting adenomas. All postoperative samples were immediately frozen in liquid nitrogen and stored at -80° C in the refrigerator.

### Protein extraction and digestion

A total of 19 tissue samples from patients with early-stage pituitary adenomas were lysed with UA buffer (8 M urea in 0.1 M Tris-HCl, pH 8.5), and sonicated on ice (180 W, 1 second on and 2 seconds off, for 99 cycles). The lysate was centrifuged at 14,000 × *g* for 15 min. The supernatant was collected and quantified using the Bradford method. The protein was lysed by filter-aided sample preparation (FASP) following the protocol described in references (9, 10).

### Quantitative proteomic analysis

The peptides used for DDA analysis were pre-fractionated by high-pH reversed-phase chromatography (Hp-RP). Two hundred micrograms of peptides were loaded and separated with a 45-min gradient, pH 10.45. Fractions were collected, and 12 fractions were combined, heat-dried, and stored at -80°C after centrifugation. The LC-MS/MS detection system consisted of a nanoflow high-performance liquid chromatography (HPLC) instrument (Easy-nLC 1000 System; Thermo Fisher, Waltham, MA, USA) coupled to a Q-Exactive HF mass spectrometer (Thermo Fisher). A home-packing column (150-μm inner diameter, ReproSil-Pur C18-AQ, 1.9 μm; Dr. Maisch) with a length of 20 cm was used for peptide separation at 60°C. The flow rate was 600 nL/min over a 90-min gradient (0–8 min, 3–8% B; 8–68 min, 8–20% B; 68–83 min, 20–30% B; 83–84min, 30–90% B; 84–90 min, 90% B). The full MS scan range was 400–1200 Da. MS/MS was operated in the top 20 modes. iRT (Biognosys, Schlieren, Switzerland) was added as an internal standard based on the manufacturer’s instructions. Peptides from 293 cell lysis were used in all experiments. The LC-MS/MS system for the DIA analysis consisted of a nanoflow HPLC (Easy-nLC 1000 System; Thermo Fisher) coupled to a Q-Exactive HF mass spectrometer (Thermo Fisher). The flow rate was 600 nL/min over different gradients. The range of the MS1 survey scan was 400–1200 m/z, followed by 29 MS2 scans of overlapping sequential precursor isolation windows (20 m/z isolation width, 40 m/z isolation width, 50 m/z isolation width, and 1 m/z overlap). The accumulation time was set according to the experimental requirements. The chromatograph peak width was 18 s. iRT was added to ensure calibration on difficult matrices, allowing for detailed quality control. All data were obtained on a 20-cm analytical column. DDA raw data files were searched against the human UniProt database (20190617 with sequences of iRT peptides) using MaxQuant (version 1.6.5.0) with its default settings. The false discovery rate (FDR) was set to 0.01 for both peptides and proteins. The DDA data search results were imported into Spectronaut (version 14) with its default settings. The FDR threshold was set to less than 0.01. The DIA raw data files were directly imported into Spectronaut with its default settings, and the results for protein identification and quantitation were exported using protein and peptide FDRs of less than 0.01.

### Differential protein acquisition and enrichment analysis

Quantitative difference analysis was performed using the quantitative protein values. For a two-by-two comparison between invasive group B and non-invasive group A, the mean value of the signals in each group was calculated, from which the ratio between groups was calculated. The comparison was done using the student’s t-test. Proteins that met the following conditions were screened as differentially expressed proteins (DEPs) (1): Ratio between groups >=2 or <=0.5; (2) P-value <0.05. The Volcano plot was drawn using DEPs to show the difference between the groups. In the volcano plot, the vertical axis shows -log10(p-value) and the horizontal axis shows log2(Fold change). Upregulated DEPs with significant fold change and p-value are shown by red dots. Downregulated DEPs are shown by green dots, and other proteins are shown by black dots. Unsupervised hierarchical cluster analysis was performed for DEPs to show the expression of DEPs in each sample in the two groups. Finally, we performed GO (Biological Process, Molecular Function, Cellular Component) enrichment analysis and KEGG pathway enrichment analysis on the DEPs. We performed bioinformatic annotation and analysis at two levels to analyze the functional properties of these proteins and the corresponding signaling pathways to select key proteins for further research.

### EMT-related DEPs identification

We screened EMT-related proteins using the GeneCards database (http://www.genecards.org/), which integrates genomic, transcriptomic, proteomic, clinical, and other relevant information related to genes. We collected and collated data from over 100 sites. The screening criteria were correlation score ≥ 3. Then, the Venn program (http://bioinformatics.psb.ugent.be/webtools/Venn/) was used to identify the intersection between EMT-related proteins and DEPs, as EMT-DEPs. To explore the interactions between EMT-DEPs, we entered EMT-DEPs into the STRING database (https://string-db.org/) to obtain the protein interaction network. To find the most important pathways, we performed GO functional annotation and KEGG pathway enrichment analysis of EMT-DEPs using R software to obtain their potential protein functions and key pathways.

### Hub EMT-related DEPs

To identify hub proteins in EMT-DEPs. We imported the information on EMT-DEPs interaction into Cytoscape software and screened hub proteins using the cytoHubba plugin. The cytoHubba plugin can predict and explore the key nodes and sub-networks in a given network by several topological algorithms. We calculated the rankings using the MCC algorithm and selected the top 20 EMT-DEPs as the hub EMT-DEPs. The protein interaction network of hub EMT-DEPs was visualized by Cytoscape software to show their potential interactions.

### Core EMT-related DEPs

To assess the correlation between EMT-DEPs, we performed a correlation analysis between EMT-DEPs to determine the paired proteins of EMT-DEPs (Cor>0.7, P<0.05). The EMT-related DEPs were ranked by the number of paired proteins, and the top 20 were identified as core EMT-related DEPs. We then used the GeneMANIA database to map the protein network of core EMT-related DEPs and their associated proteins. The GeneMANIA database (http://genemania.org/) was used to generate hypotheses about gene function and to find genomic and proteomic data regarding the function of target proteins.

### Analysis and validation of Key EMT-related DEPs

We identified the intersection of hub EMT-related DEPs and core EMT-related DEPs through the Venn online program as the key EMT-related DEPs. The R software was used to analyze the correlation between the quantitative and clinical features of key EMT-related DEPs. The ROC curve was used to evaluate the sensitivity and accuracy of key EMT-related DEPs in predicting the invasion of pituitary adenomas. We also used the GSE169498 gene expression profile to measure clinical correlation. ROC curve analysis was used to assess the efficacy of key EMT-related DEPs expression in predicting the invasive behavior of pituitary adenoma. Wilcoxon signed-rank test was used to evaluate the relationship between the groups. P<0.05 was considered statistically significant.

### Potential function and EMT relevance of the target proteins

We identified the target proteins using proteomics analysis and GSE169498 chip. Then, the paired proteins of the target protein were screened (Cor>0.7, P<0.05), and the correlation between them was measured by R software. We input the target protein and paired proteins into the GeneMANIA database to explore the potential functions of the target proteins and related key pathways. GO functional annotation and KEGG pathway enrichment of target and paired proteins were performed to identify the role of the target proteins in the invasion and EMT of pituitary adenomas and their related key pathways. To verify the relevance of the target proteins to EMT, we verified the co-expression of target proteins and classical EMT markers (CDH1, CDH2, DSP, FN1, ITGB6, MMP9, TJP1, and VIM) by quantitative proteomic correlation analysis. We found which markers of EMT are related to target proteins.

### Experimental validation of target protein

This study was approved by the Institution Review Board of the Beijing Tiantan Hospital. Between December 2020 and November 2021, specimens were obtained from patients who underwent surgery at Beijing Tiantan Hospital, Beijing, China. Informed consent was obtained from all individuals. All patients provided written informed consent. Each sample was allocated to the invasive or non-invasive group based on the Knosp grade. We collected the clinical information of patients. All samples were fixed in 10% formalin for 24 h, paraffin-embedded, and then cut into 5 μm thick sections. Glass slide was inserted, and the samples were dewaxed and rehydrated. After antigen repair and endogenous peroxidase blocking, goat serum was used to seal. Then, diluted antibodies were added and incubated at 4°C overnight. After washing with PBS, enzyme-labeled IgG polymer was added and incubated at room temperature for 20 min. Finally, diaminobenzidine (DAB) color development solution and hematoxylin were used as the double stain to visualize the antibodies. The negative control was stained with PBS buffer instead of antibodies, and the known positive tissue (breast) was used as positive control. Images were taken using a slide scanner (Leica, Germany). Two experienced neuropathologists independently assessed the samples. The percentage of positive cells was calculated under high magnification (×400).

## Results

### DEPs and enrichment analysis

**Figure 2:**
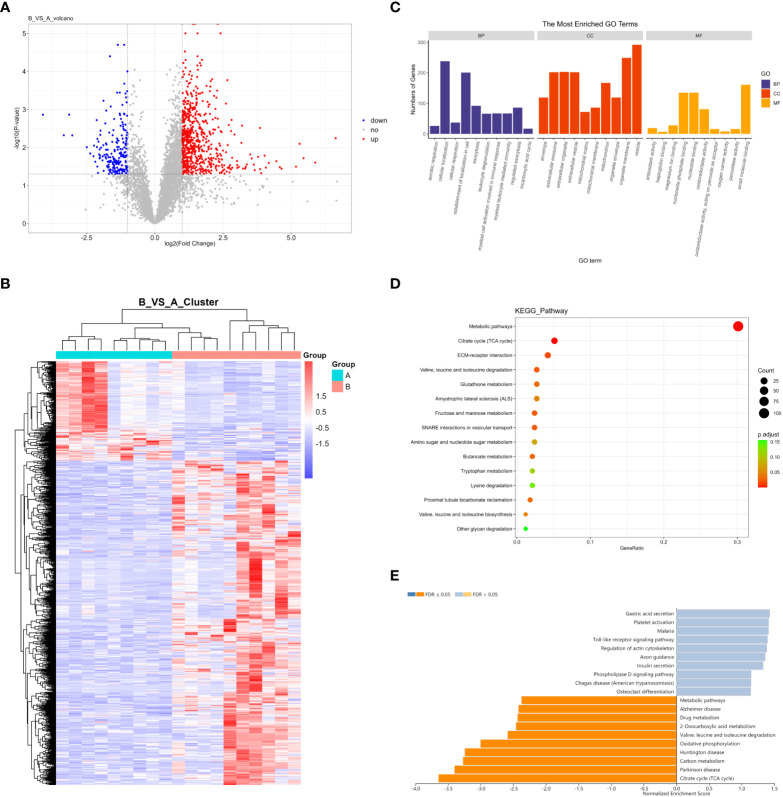
**(A)** Volcano map of DEPs; **(B)** Cluster analysis heat map of DEPs; **(C)** GO enrichment analysis of DEPs; **(D)** KEGG enrichment analysis of DEPs; **(E)** GSEA enrichment analysis of DEPs.

In total, 5598 proteins were identified in the quantitative analysis of proteomics data, and 833 DEPs were obtained after differential analysis. Among them, 638 were upregulated and 195 were downregulated in invasive pituitary adenomas (**Figure 2A**). Cluster analysis of DEPs revealed that there were significantly more upregulated proteins in the invasive group and more downregulated proteins in the non-invasive group (**Figure 2B**). The GO function and KEGG pathway enrichment analysis of DEPs showed that: the BP functions enriched with DEPs included cellular localization, establishment of localization in cell, and CC functions included organelle membrane, vesicle, MF functions included nucleoside phosphate binding, nucleotide binding, and small-molecule binding (**Figure 2C**). Metabolic pathways, citrate cycle (TCA cycle), and ECM−receptor interaction were the major enrichment pathways of DEPs (**Figure 2D**). GSEA enrichment analysis showed that the main pathways affected by DEPs were citrate cycle (TCA cycle), Parkinson’s disease, and carbon metabolism (**Figure 2E**).

### EMT-related DEPs

**Figure 3:**
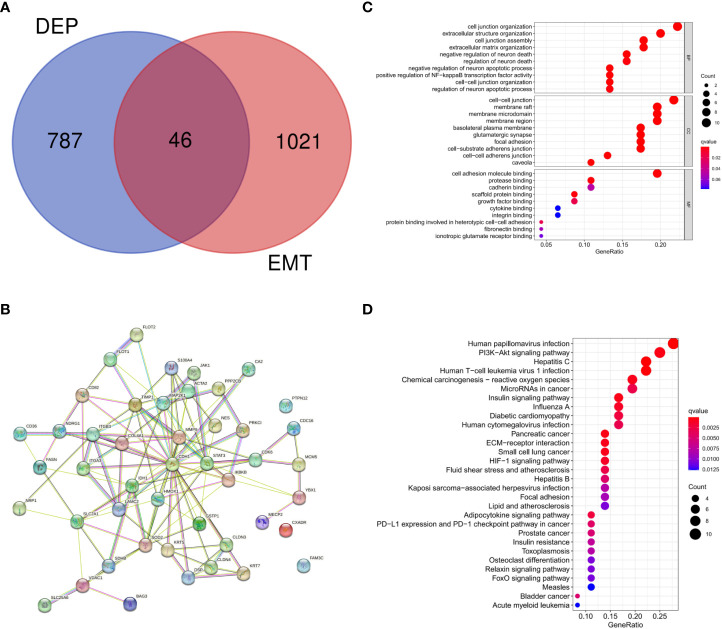
**(A)** Intersection of DEPs and EMT-related proteins; **(B)** Protein interaction network of EMT-DEPs; **(C)** GO enrichment analysis of EMT-DEPs; **(D)** KEGG enrichment analysis of EMT-DEPs.

There were 46 intersections between EMT-related proteins and DEPs in the GeneCards database (**Figure 3A**). The STRING database visualized potential protein interactions between EMT-DEPs (**Figure 3B**). Potential GO functions of EMT-DEPs at BP level included: cell junction organization, extracellular structure organization, cell junction assembly, and extracellular matrix organization. Potential GO functions of EMT-DEPs at CC level included: cell−cell junction, membrane raft, membrane microdomain, and membrane region. Potential GO functions of EMT-DEPs at MF level included: cell adhesion molecule binding, protease binding, cadherin binding, and scaffold protein binding (**Figure 3C**). KEGG pathway enrichment analysis showed that EMT-related DEPs were mainly enriched in human papillomavirus infection, PI3K/Akt signaling pathway, hepatitis C infection, human T−cell leukemia virus 1 infection, chemical carcinogenesis−reactive oxygen species, microRNAs in cancer, and insulin signaling pathway (**Figure 3D**).

### Hub EMT-related DEPs and core EMT-related DEPs

**Figure 4:**
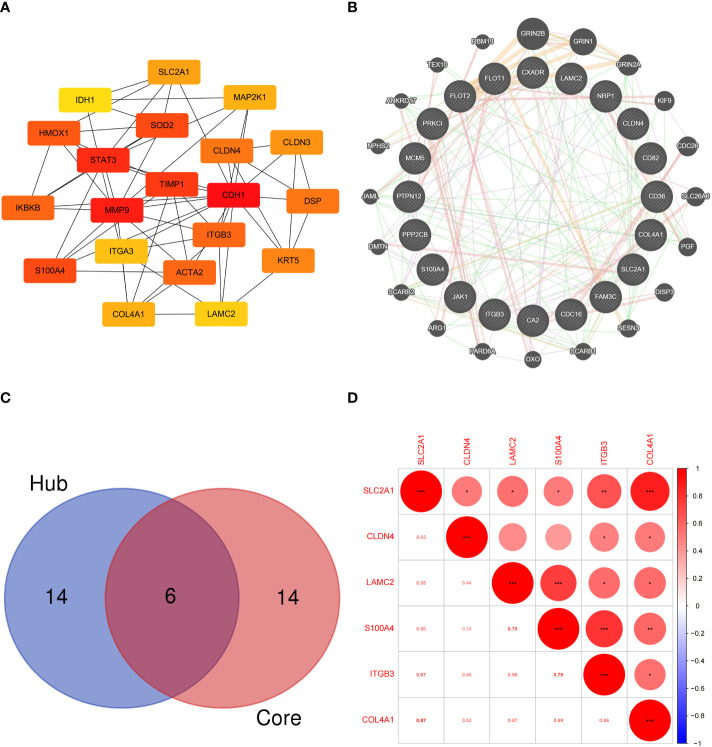
**(A)** Protein interaction network of hub EMT-related DEPs; **(B)** Interaction network of core EMT-related DEPs and related proteins in the GeneMANIA database; **(C)** Intersection of the hub EMT-related DEPs and core EMT-related DEPs; **(D)** Correlation analysis of key EMT-related DEPs expression in proteomics.

The hub EMT-related DEPs were identified in Cytoscape using the cytoHubba plugin. The MCC scores were ranked. The top 20 positions were CDH1, MMP9, STAT3, TIMP1, S100A4, SOD2, HMOX1, ACTA2, ITGB3, IKBKB, DSP, CLDN4, KRT5, CLDN3, SLC2A1, COL4A1, MAP2K1, ITGA3, LAMC2, and IDH1 (**Table 1**). Their potential protein interactions are shown in **Figure 4A**. Based on the pairwise correlation analysis of 46 EMT-related DEPs by R software, the top 20 core EMT-DEPs with the highest number of correlated paired proteins, were FLOT1, FLOT2, CD36, PPP2CB, PTPN12, CA2, COL4A1, NRP1, MCM5, CD82, SLC2A1, CLDN4 CXADR, FAM3C, ITGB3, PRKCI, JAK1, LAMC2, S100A4, and CDC16, respectively (**Table 2**). The GeneMANIA database exhibits their interactions and related genes (**Figure 4B**).

**Table 1:** Top 20 hub EMT-related DEPs ranked by MCC score.

PG.Genes	PG.Protein Descriptions	MCC score
CDH1	Cadherin 1	142
MMP9	Matrix Metallopeptidase 9	122
STAT3	Signal Transducer And Activator Of Transcription 3	94
TIMP1	TIMP Metallopeptidase Inhibitor 1	74
S100A4	S100 Calcium Binding Protein A4	48
SOD2	Superoxide Dismutase 2	48
HMOX1	Heme Oxygenase 1	42
ACTA2	Actin Alpha 2, Smooth Muscle	32
ITGB3	Integrin Subunit Beta 3	32
IKBKB	Inhibitor Of Nuclear Factor Kappa B Kinase Subunit Beta	32
DSP	Desmoplakin	30
CLDN4	Claudin-4	30
KRT5	Keratin 5	26
CLDN3	Claudin-3	24
SLC2A1	Solute Carrier Family 2 Member 1	23
COL4A1	Collagen Type IV Alpha 1 Chain	18
MAP2K1	Mitogen-Activated Protein Kinase Kinase 1	18
ITGA3	Integrin Subunit Alpha 3	15
LAMC2	Laminin Subunit Gamma 2	14
IDH1	Isocitrate Dehydrogenase (NADP(+)) 1	12

**Table 2:** Top 20 core EMT-related DEPs based on the number of paired proteins.

PG.Genes	PG.Protein Descriptions	Paired genes number
FLOT1	Flotillin-1	20
FLOT2	Flotillin-2	20
CD36	CD36 Molecule	16
PPP2CB	Protein Phosphatase 2 Catalytic Subunit Beta	13
PTPN12	Protein Tyrosine Phosphatase Non-Receptor Type 12	13
CA2	Carbonic Anhydrase 2	12
COL4A1	Collagen Type IV Alpha 1 Chain	11
NRP1	Neuropilin-1	11
MCM5	Minichromosome Maintenance Complex Component 5	10
CD82	CD82 Molecule	9
SLC2A1	Solute Carrier Family 2 Member 1	9
CLDN4	Claudin-4	8
CXADR	Coxsackievirus and Adenovirus Receptor	8
FAM3C	FAM3 Metabolism Regulating Signaling Molecule C	8
ITGB3	Integrin Subunit Beta 3	8
PRKCI	Protein Kinase C Iota	8
JAK1	Janus Kinase 1	7
LAMC2	Laminin Subunit Gamma 2	7
S100A4	S100 Calcium Binding Protein A4	7
CDC16	Cell Division Cycle 16	6

### Validation of key EMT-related DEPs

**Figure 5:**
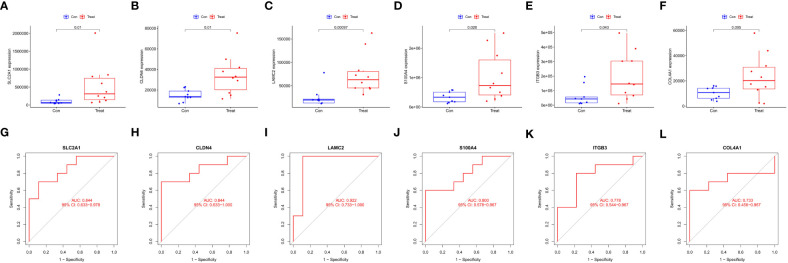
Correlation analysis between key EMT-related DEPs expression and invasiveness in proteomics: **(A)** SLC2A1; **(B)** CLDN4; **(C)** LAMC2; **(D)** S100A4; **(E)** ITGB3; **(F)** COL4A1. ROC curve of key EMT-related DEPs predicting invasiveness in proteomics: **(G)** SLC2A1; **(H)** CLDN4; **(I)** LAMC2; **(J)** S100A4; **(K)** ITGB3; **(L)** COL4A1.

**Figure 6:**
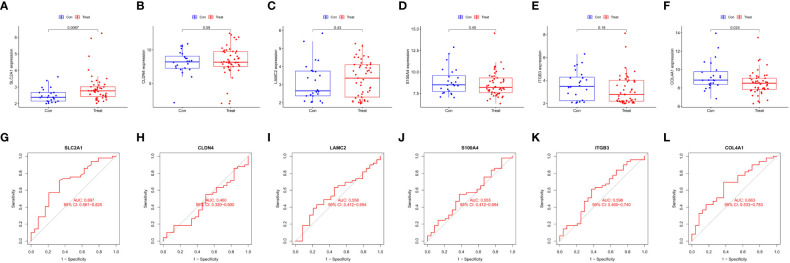
Correlation analysis of Key EMT-DEPs expression and invasiveness in GSE169498: **(A)** SLC2A1; **(B)** CLDN4; **(C)** LAMC2; **(D)** S100A4; **(E)** ITGB3; **(F)** COL4A1. ROC curve of Key EMT-DEPs predicting invasivity in GSE169498: **(G)** SLC2A1; **(H)** CLDN4; **(I)** LAMC2; **(J)** S100A4; **(K)** ITGB3; **(L)** COL4A1.

Six intersecting key EMT-related DEPs, including SLC2A1, CLDN4, LAMC2, S100A4, ITGB3, and COL4A1, were identified from the hub EMT-related DEPs and core EMT-related DEPs using Venn online program (**Figure 4C**). A significant positive correlation was found between all of them according to the quantitative protein co-expression correlation (**Figure 4D**, cor>0.3). SLC2A1, CLDN4, LAMC2, S100A4, and ITGB3 were significantly correlated with the invasiveness of pituitary adenoma (P<0.05, **Figures 5A–E**). COL4A1 was not significantly correlated with the invasiveness of pituitary adenoma (P>0.05, **Figure 5F**). The areas under the ROC curves of the six intersection proteins for predicting invasion were greater than 0.7 (**Figures 5G–L**). SLC2A1 was significantly associated with the invasiveness of pituitary adenoma in the GSE169498 microarray. Consistent with the results of protein quantification (P<0.05, **Figure 6A**), SLC2A1 was significantly upregulated in the invasive group. Contrary to the results of proteomic quantification, COL4A1 was downregulated in invasive pituitary adenomas (P<0.05, **Figure 6F**). CLDN4, LAMC2, S100A4, and ITGB3 were not significantly associated with the invasiveness of pituitary adenoma (P>0.05, **Figures 6B–E**). The area under the ROC curve for predicting invasion by SLC2A1 was 0.697 (**Figure 6G**), and the AUC values for the remaining genes were <0.65 (**Figures 6H–L**).

### Target protein identification

**Figure 7:**
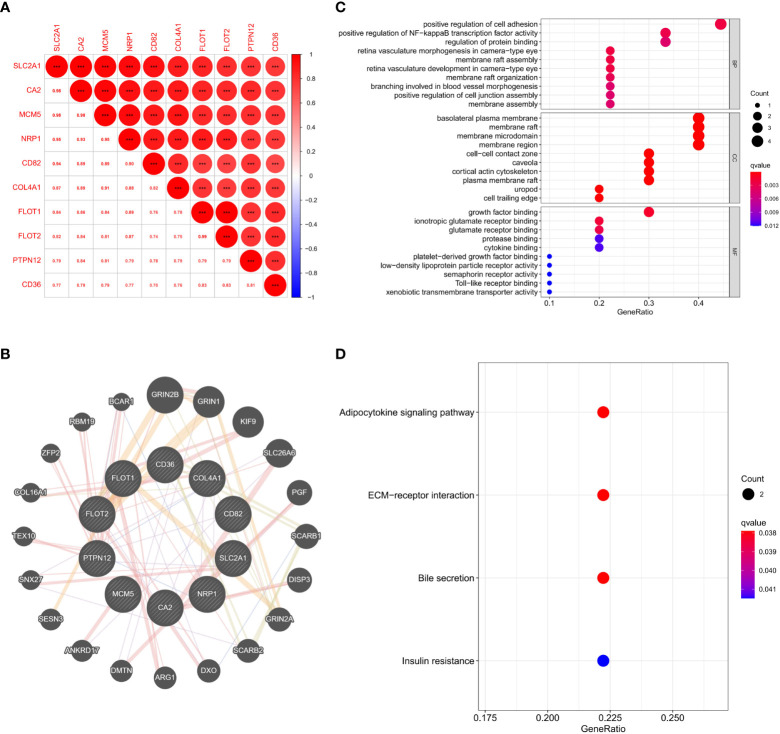
**(A)** Expression correlation analysis of SLC2A1 and its paired proteins in proteomics; **(B)** Protein interaction network of SLC2A1 and its paired proteins in the GeneMANIA database; **(C)** GO enrichment analysis of SLC2A1 and its paired proteins; **(D)** KEGG enrichment analysis of SLC2A1 and its paired proteins.

As SLC2A1 was similarly correlated with invasion in proteomics and GSE169498 microarrays, it may be a potential marker for invasion in pituitary adenoma. We demonstrated the co-expression correlation of SLC2A1 with its paired proteins (CA2, MCM5, NRP1, CD82, COL4A1, FLOT1, FLOT2, PTPN12, and CD36) (**Figure 7A**). Because of their high correlation, we mapped the protein network of SLC2A1 with its paired proteins. SLC2A1 and its paired proteins were related to GRIN2B, GRIN1, KIF9, SLC26A6, PGF, SCARB1, DISP3, GRIN2A, SCARB2, DXO, ARG1, DMTN, ANKRD17, SESN3, SNX27, TEX10, COL16A1, ZFP2, RBM19, and BCAR1 (**Figure 7B**). GO and KEGG enrichment analysis showed that SLC2A1 and its paired proteins (CA2, MCM5, NRP1, CD82, COL4A1, FLOT1, FLOT2, PTPN12, and CD36) were mainly enriched in positive regulation of cell adhesion, positive regulation of NF−kappa B transcription factor activity, regulation of protein binding (BP function), basolateral plasma membrane, membrane raft, membrane microdomain, membrane region, cell−cell contact zone (CC function), growth factor binding, ionotropic glutamate receptor binding, and glutamate receptor binding (MF function) (**Figure 7C**). Their main enriched pathways included adipocytokine signaling pathway, ECM−receptor interaction, bile secretion, and insulin resistance (**Figure 7D**).

### Target protein with EMT

**Figure 8:**
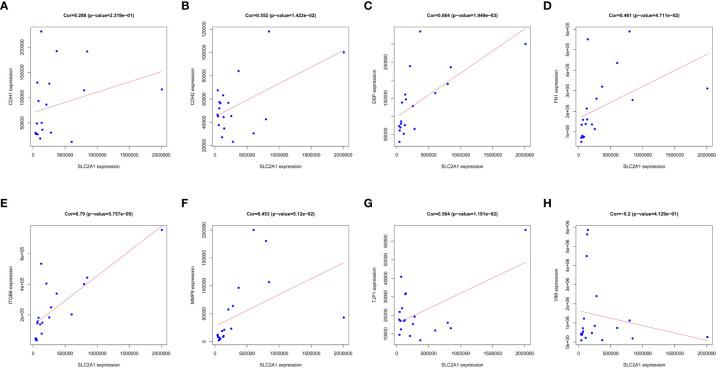
Correlation between SLC2A1 expression and EMT markers in proteomics: **(A)** CDH1; **(B)** CDH2; **(C)** DSP; **(D)** FN1; **(E)** ITGB6; **(F)** MMP9; **(G)** TJP1; **(H)** VIM.

We analyzed the key factors affecting the association of SLC2A1 with EMT. We performed co-expression correlation analysis between SLC2A1 and classical EMT markers (CDH1, CDH2, DSP, FN1, ITGB6, MMP9, TJP1, and VIM) using proteomic quantification. We found a significant positive expression correlation of SLC2A1 with CDH2, DSP, FN1, ITGB6, and TJP1 (Cor>0.3, P<0.05, **Figures 8B–E, G**). In contrast, there was no significant correlation between SLC2A1 and CDH1, MMP9, or VIM (P>0.05, **Figures 8A, F, H**).

### Experimental validation of the target protein

**Figure 9:**
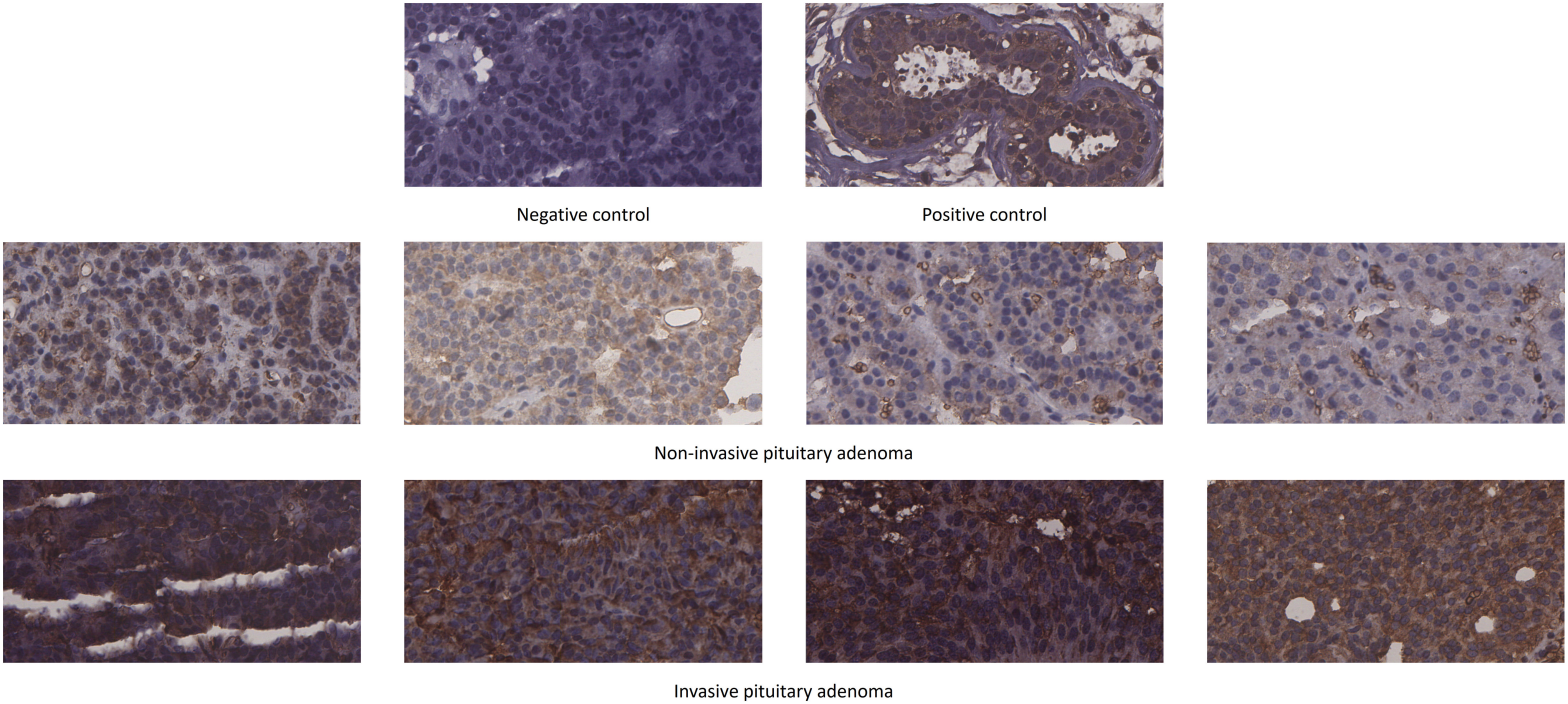
Immunohistochemical staining of SLC2A1 in non-invasive and invasive pituitary adenomas.

We used immunohistochemical staining to detect SLC2A1 expression in non-invasive and invasive pituitary adenomas. The results showed that SLC2A1 was mainly expressed in the cytoplasm. SLC2A1 expression was significantly higher in invasive pituitary adenomas than in non-invasive pituitary adenomas, the difference was statistically significant (P<0.05) (**Figure 9**). This was consistent with our quantitative proteomics and GSE169498 analysis, suggesting that SLC2A1 may be a potential biomarker of invasion for pituitary adenoma.

## Discussion

Invasive pituitary adenomas usually invade the surrounding normal tissues, which complicates surgical resection and increases the risk of recurrence. Therefore, patients often need hormone therapy, radiotherapy, and chemotherapy after surgery (11, 12). The entire process requires multidisciplinary cooperation between endocrinologists, neurosurgeons, radiation therapists, and oncologists. Exploring the underlying mechanisms involved in the development, invasion, and metastasis of pituitary adenoma can help prevent tumor progression. EMT is crucial for tumor invasion and metastasis. It has been shown that EMT may be associated with the invasiveness of pituitary adenomas; thus, it is necessary to identify the EMT markers associated with invasion in pituitary adenomas. Using quantitative and differential proteomics analysis, we identified proteins associated with the invasion of pituitary adenomas. We combined them with EMT-associated proteins and screened key aggression markers significantly associated with EMT in pituitary adenomas. Finally, we identified SLC2A1 as the target biomarker. Then, the linkage between SLC2A1 and EMT was unveiled by enrichment analysis and EMT marker correlation analysis. In addition, the differential expression of SLC2A1 in invasive pituitary adenomas was experimentally verified.

SLC2A1 (also known as GLUT1, Glucose transporter 1) is a rate-limiting factor for glucose uptake, which contributes to insulin-independent glucose uptake. The unlimited capacity of tumor cells for growth and proliferation requires an adequate amount of energy; therefore, SLC2A1 is upregulated in many tumors (13–15). It has been found that GLUT1 can promote cancer cell proliferation, invasion, and migration. High expression of GLUT1 is associated with poor prognosis in many solid tumors (14). In our study, SLC2A1 was significantly overexpressed in invasive pituitary adenomas, but the exact mechanism by which SLC2A1 promotes the progression of pituitary adenomas remains unknown. This may be due to the presence of a hypoxic and acidic microenvironment in solid tumors, which inhibits oxidative phosphorylation and enhances aerobic glycolysis, thereby reprogramming energy metabolism in tumor cells (16). Glucose is the main source of energy for metabolism. Tumor cells can survive and proliferate by reprogramming their metabolism (17, 18). The main features of this metabolism reprogramming are increased glucose uptake and the conversion of glucose to lactate. Tumor cells increase glucose consumption and convert glucose to lactate even in the presence of a sufficient amount of oxygen. Tumor cells prefer anaerobic glycolysis over mitochondrial oxidative phosphorylation (OXPHOS), a phenomenon known as the Warburg effect or aerobic glycolysis (19, 20). Compared to OXPHOS, glycolysis produces ATP faster but less efficiently. This inefficiency can be compensated by increased glucose uptake through transmembrane glucose transporter protein (GLUT) (21). Therefore, tumor cells usually exhibit elevated glucose metabolism, and increased glucose uptake (22, 23), which can support rapid ATP production and tumor progression.

It has been suggested that GLUTs may promote cancer development by activating NF-κB, PI3K/Akt pathway, and wild-type p53 protein expression (24–28). Fourteen members of the GLUT protein family have been identified in humans, of which GLUT1, GLUT3, and GLUT4 have the highest affinity for glucose (20, 29). GLUT1 overexpression in tumor cells can significantly contribute to tumor growth through the Warburg effect. SLC2A1 inhibits oxidative phosphorylation and enhances cellular glycolysis, helping tumor cells to adapt to their hypoxic microenvironment (18, 30). Increased glycolysis and overproduction of lactic acid in tumor cells decrease pH in the tumor microenvironment. The acidic microenvironment kills the surrounding normal cells, and leads to the release of protein hydrolases and consequent remodeling of extracellular matrix (ECM) (31–33), and a significant depletion of intercellular adhesion proteins (e.g. E-calcine mucin, E-CAD) (34). Our study showed that SLC2A1 may affect the positive regulation of cell adhesion, regulation of protein binding, basolateral plasma membrane, cell-cell contact zone, growth factor binding, ECM-receptor interaction, and other processes. All of these may promote epithelial-to-mesenchymal transition (EMT), thereby promoting cancer cell migration and invasion (35). In addition, lower amounts of reactive oxygen species (ROS) are produced due to reduced OXPHOS and oxygen consumption, which may also promote cancer cell proliferation and prevent apoptosis (36).

In addition, GLUT1 can induce epithelial-mesenchymal transition (EMT) by regulating matrix metalloproteinase (MMP) activity to enhance cell invasion and metastasis (37, 38). Matrix metalloproteinases are critical for invasion and metastasis of malignant tumor cells. Among them, MMP-2 and MMP-9 are associated with the malignant phenotype because of their ability to degrade type IV collagen in the basement membrane (39–41). In our quantitative proteomic correlation analysis, there was a significant positive correlation between SLC2A1 expression and CDH2 (N-cad), DSP, FN1, ITGB6, and TJP1, but there was no significant correlation with CDH1 (E-cad), MMP9, and VIM expression. During EMT, epithelial cells acquire mesenchymal phenotype. After EMT, epithelial cells lose their intercellular adhesion and polarity (42, 43). Thus, EMT enables tumor cells to leave the primary tissue and accelerates distant metastasis. EMT allows tumor cells to invade the surrounding tissues and eventually metastasize to distant sites (44). TWIST family bHLH transcription factor 1 (TWIST1) is one of the basic regulators of EMT. It is a proto-oncogene regulated by AKT signaling (45, 46). TWIST1 enhances glucose uptake by upregulating GLUT1, thereby reprogramming glucose metabolism in tumor cells (46, 47). Li et al. found that GLUT1 overexpression is associated with increased glucose uptake during EMT. Herein, Zhang et al. demonstrated that EMT was accompanied by upregulation of GLUT1 in an osteosarcoma cell line, and EMT was significantly inhibited after GLUT1 knockdown (48). GULT1 was positively correlated with EMT-related proteins, Vim and N-cad, and negatively correlated with E-cad during laryngeal cancer cell invasion and metastasis (49). Similar findings were reported by Mayer et al. (50). In summary, we suggest that GLUT1 overexpression may induce EMT, thereby promoting invasion and metastasis in pituitary adenomas.

## Conclusion

We performed a quantitative proteomic comparison between invasive and non-invasive pituitary adenomas, using a series of analytical approaches to identify DEPs most relevant to EMT and pituitary adenomas invasiveness. Ultimately, we identified SLC2A1 as an EMT-related DEP. Proteomics data and experiments verified that SLC2A1 was significantly upregulated in invasive pituitary adenomas. The analysis also showed that SLC2A1 and its paired proteins may affect ECM-receptor interaction. There was a positive co-expression correlation between SLC2A1 and EMT-related markers. In conclusion, SLC2A1 expression is associated with the invasiveness of pituitary adenoma. SLC2A1 may regulate EMT. SLC2A1 is a potential biomarker and therapeutic target for invasion of pituitary adenomas. These findings can guide future studies on invasive pituitary adenomas and provides a theoretical basis for clinical practice. We will continue to explore the mechanisms related to the invasion and progression of pituitary adenoma.

## Data availability statement

Publicly available datasets were analyzed in this study. This data can be found here: https://www.proteomexchange.org/, PXD039328; https://www.ncbi.nlm.nih.gov/geo/, GSE169498.

## Ethics statement

The studies involving human participants were reviewed and approved by the ethics committee of Beijing Tiantan Hospital, Capital Medical University. The patients/participants provided their written informed consent to participate in this study.

## Author contributions

YZ, LL and XM were jointly responsible for the analysis of the data and writing of the paper. CL and GL participated in the linguistic polishing of the manuscript. ZB helped search the literature, while ZY was responsible for data collation. PL was responsible for reviewing improvements and financial support. All authors contributed to the article and approved the submitted version.
