# Supplementary figures and images for: Quantitative proteomics identified a novel invasion biomarker associated with EMT in pituitary adenomas
Source: Front Endocrinol (Lausanne). 2023 Mar 3;14:1137648. doi: 10.3389/fendo.2023.1137648 (PMC10020714; doi:10.3389/fendo.2023.1137648)

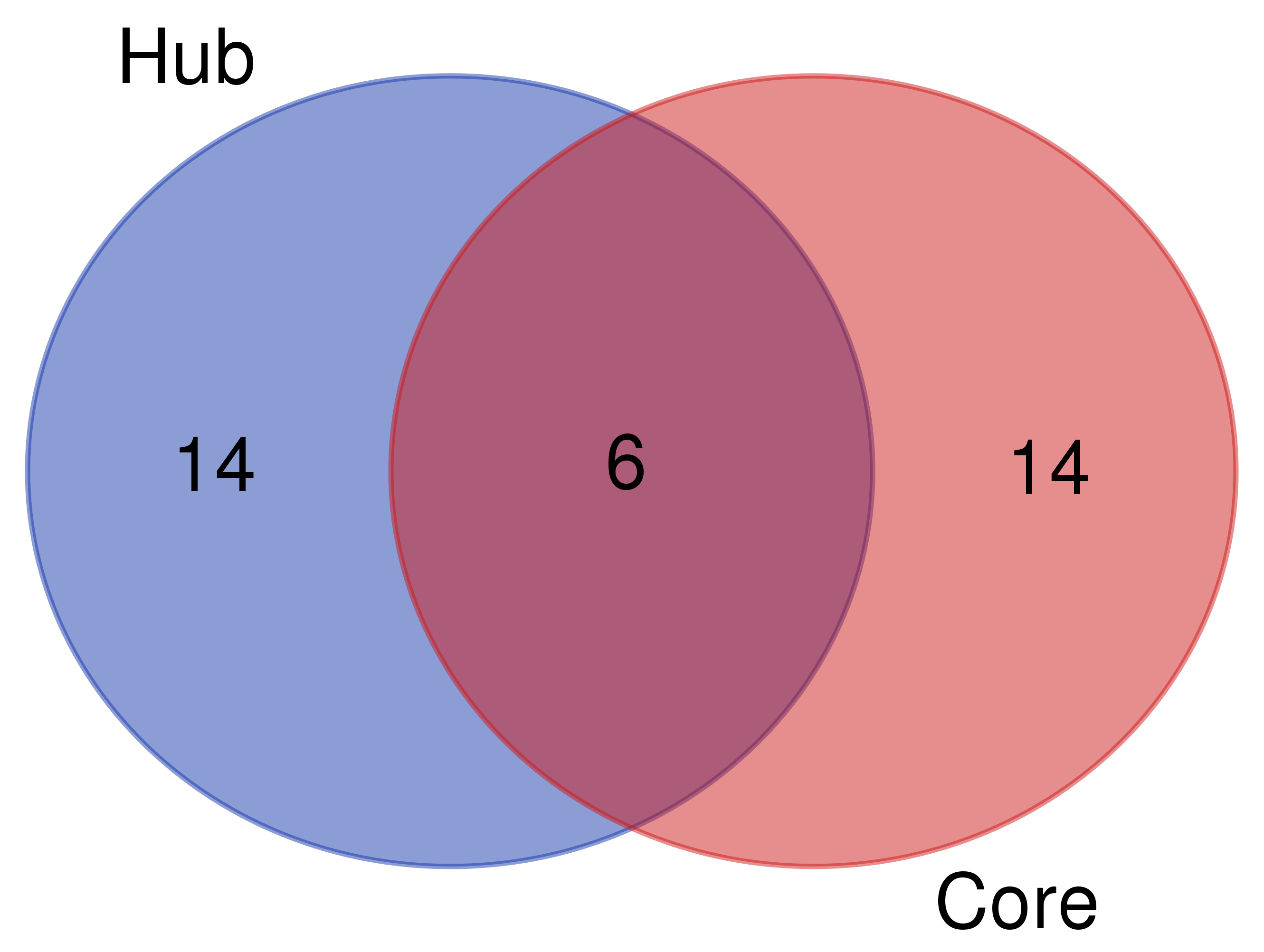

Supplement: Supplementary file 1 [file DataSheet_1.zip › source data/venn_result32694.png]

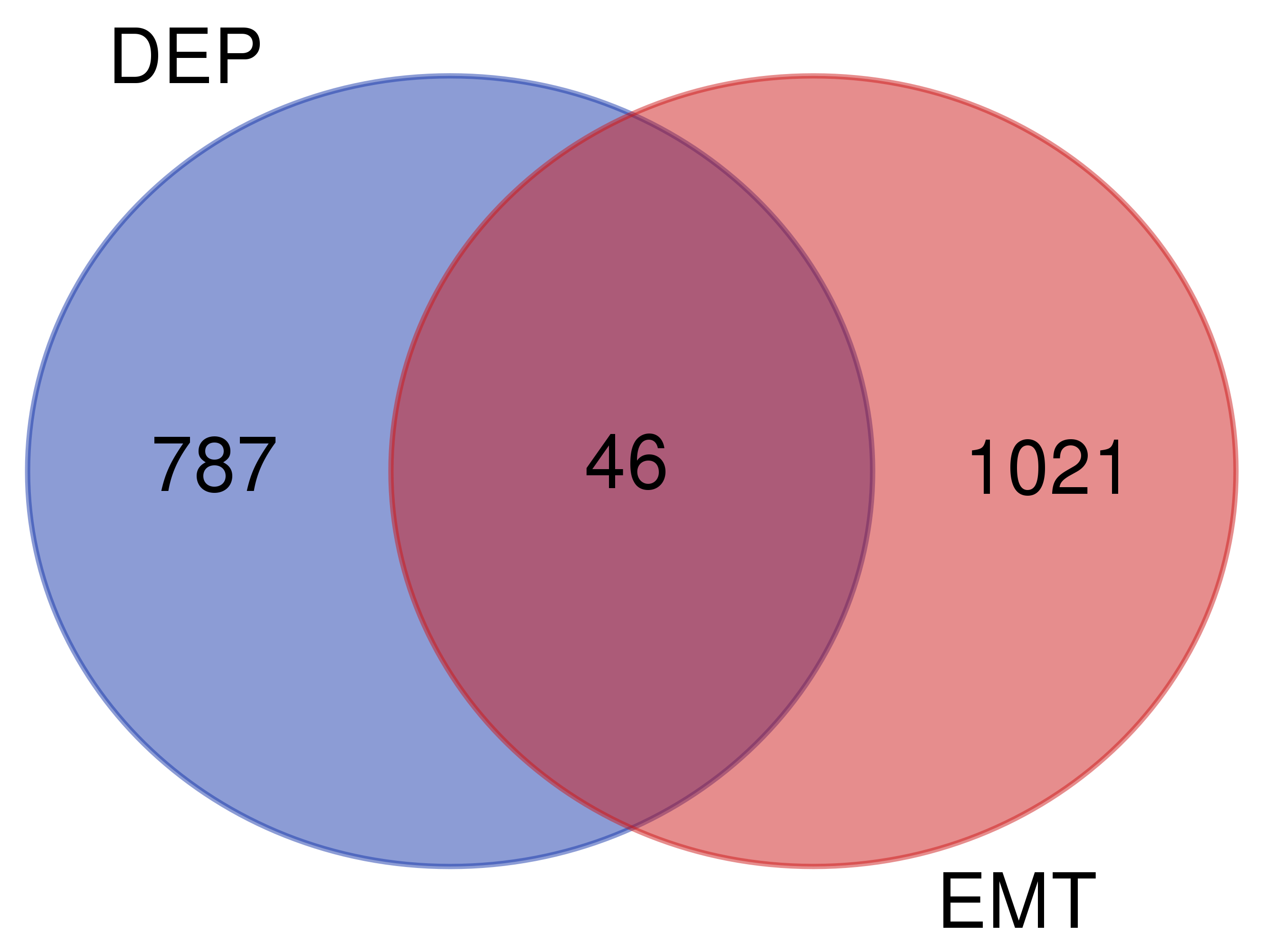

Supplement: Supplementary file 1 [file DataSheet_1.zip › source data/venn_result7754.png]
